# Pedicled Functional Latissimus Flaps for Reconstruction of the Upper Extremity following Resection of Soft-Tissue Sarcomas

**DOI:** 10.3390/curroncol30030237

**Published:** 2023-03-07

**Authors:** Alexandra M. Arguello, Mikaela H. Sullivan, Gavin L. Mills, Steven L. Moran, Matthew T. Houdek

**Affiliations:** 1 Mayo Clinic Department of Orthopedic Surgery, Rochester, MN 55905, USA; 2 Mayo Clinic Division of Plastic and Reconstructive Surgery, Rochester, MN 55905, USA

**Keywords:** upper extremity, soft tissue sarcoma, latissimus flap, functional reconstructions

## Abstract

(1) Background: Resection of soft-tissue sarcomas (STS) of the upper extremity can result in substantial functional impairment with limited options for functional reconstruction. Free functional latissimus flaps have been utilized to restore function of the thigh; however, there is limited data on the use of latissimus flaps for functional reconstruction in the upper extremity. As such, we sought to evaluate our institutional experience with these flaps. (2) Methods: We reviewed ten (seven male; three female; and a mean age of 63 years) patients undergoing soft-tissue sarcoma resection involving the triceps (*n* = 4), biceps (*n* = 4), and deltoid (*n* = 2) reconstructed with a pedicled functional latissimus flap. All surviving patients had at least 1 year of follow-up, with a mean follow-up of 5 years. (3) Results: The mean elbow range of motion and shoulder elevation were 105° and 150°. The mean Musculoskeletal Tumor Society score was 88%, and the muscle strength was four. Four patients had a recipient site wound complication. There were no flap losses. One patient sustained a radiation-associated humerus fracture 5 years postoperatively, treated nonoperatively. (4) Conclusions: Although early complications are high, pedicled functional latissimus flaps allow for wound coverage, potential space obliteration, and restoration of function in the upper extremity following resection of large soft tissue sarcomas.

## 1. Introduction

Limb salvage has surpassed amputation as the treatment of choice for management of upper extremity soft tissue sarcomas (STS) given innovations in imaging, radiation, and surgical techniques [1,2,3,4]. Though recently developed limb salvage techniques have improved functional outcomes compared to amputation, resection of STS of the upper extremity commonly results in functional deficits for patients, depending on the extent of resection [5,6]. The use of both free and pedicled flaps for soft tissue coverage after resection of upper extremity STS has been well described and has been shown to contribute to the preservation of limb function [7,8,9,10]. There are limited options, however, for functional flaps of the shoulder, biceps, and triceps.

Free functional latissimus flaps have been utilized to restore function of the hamstrings, quadriceps, and gastrocnemius muscles in the lower extremity [11,12,13,14] and have been shown to restore active knee function after lower extremity STS resection [15]. The use of latissimus flaps has also been described for functional reconstruction in the upper extremity in the setting of trauma and brachial plexus injury [7,16], but has not been largely analyzed with regard to function and complications in the setting of STS resection. The purpose of this study is to evaluate the outcomes of patients undergoing reconstruction of the upper extremity utilizing a pedicled functional latissimus flap after STS resection.

## 2. Materials and Methods

**Figure 1:**
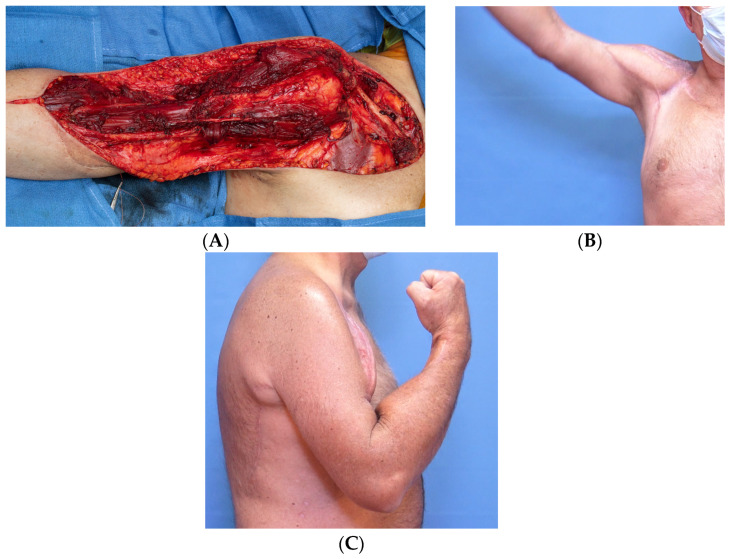
Intraoperative photograph showing resection of the entire anterior compartment of the arm (**A**). The tumor bed was reconstructed utilizing a pedicled functional latissimus flap, restoring forward elevation of the arm (**B**) and elbow flexion (**C**).

**Figure 2:**
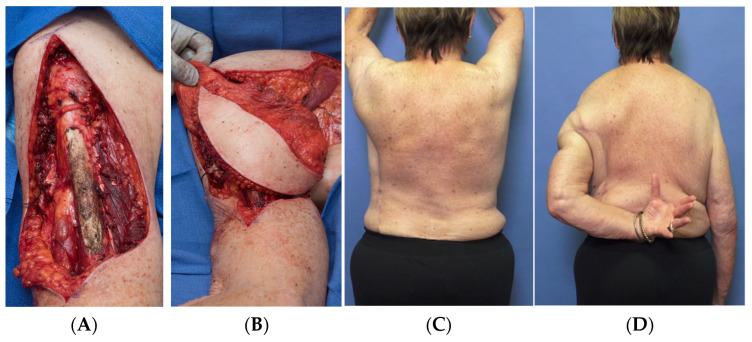
Following resection of the deltoid (**A**). The pedicled latissimus flap is elevated. (**B**) Inset to allow for active shoulder elevation. (**C**) Internal rotation (**D**).

Following institutional review board approval, we retrospectively reviewed patients who underwent resection of STS involving the upper extremity and were reconstructed with a pedicled functional latissimus flap at our institution from 2007 to 2021. The final group of patients (*n* = 10) included 7 males and 3 females with a mean age of 63 years (Table 1). The STS involved either the triceps (*n* = 4), biceps (*n* = 4, Figure 1), or deltoid (*n* = 2, Figure 2). Diagnoses included undifferentiated pleomorphic sarcoma (*n* = 4), myxofibrosarcoma (*n* = 2), pleomorphic lipoma, pleomorphic spindle cell sarcoma, Neurotrophic receptor tyrosine kinase (NTRK)-fusion associated sarcoma, and extraskeletal osteosarcoma. All tumors were high-grade except for the NTRK sarcoma.

All patients were treated with radiotherapy, neoadjuvantly in 8 cases (total dose 50 Gy) and neoadjuvantly and intraoperatively in two cases (total doses 55 and 65 Gy). The mean tumor size and volume at the time of resection were 12 cm (range, 5–24 cm) and 612 cm^3^ (range, 40–2815 cm^3^), respectively. All margins were negative at the time of the final resection.

Following surgical resection, patients were followed for complications and recurrence. Patients returned for evaluation with imaging at least every 3 months for the first 2 years, every 6 months for years 2–5, and then annually for years 5–10. Imaging included orthogonal radiographs, local cross-sectional imaging in the form of an MRI or CT, and a CT scan of the chest. All surviving patients had at least 1 year of follow-up, with a mean follow-up of 5 years (range 1–11 years).

The postoperative active range of motion of the shoulder was evaluated with active forward elevation and external rotation recorded in degrees. Functional outcomes were assessed utilizing the Musculoskeletal Tumor Society scores at the final follow-up [17].

### Surgical Technique

Resections are performed in a rolling lateral position, with harvest of the latissimus performed in a standard fashion following tumor extirpation. Typically, a curvilinear incision is used for the harvest of the latissimus flap. Perforators for a skin paddle are identified using a handheld Doppler probe prior to making the incision. All flaps were harvested with a skin paddle in this series. The resting tension of the latissimus is marked with sutures placed in 5 cm increments prior to flap elevation. The latissimus is delineated anteriorly from the serratus musculature using the different muscle fibers, medially from the paraspinal musculature, and inferiorly dissected down to the iliac crest insertion. After the whole muscle is defined at its borders, the muscle is raised off the deep musculature, proceeding from the superomedial level and down toward the medial region, then reflecting the muscle back superiorly and detaching from the serratus. All perforating branches to the paraspinal musculature and the intercostal perforators are clipped and ligated to ensure complete hemostasis. The vascular pedicle, which includes the thoracodorsal artery, vein, and accompanying nerve, is identified underneath the latissimus muscle and preserved throughout the case. The incision is either extended to the incision used for resection or a tunnel is created to pass the latissimus flap to the area requiring coverage. The muscle and skin flap are laid down in the defect, and excess muscle is trimmed away using a sharp dissection. The skin paddle is centered on the defect, with muscle on either side of the skin paddle for any required coverage of critical structures in the arm. The muscle is then attached to the cut ends of the resected tendon distally with multiple locking sutures, or transosseously in cases of deltoid reconstruction. Proximally, the latissimus is attached to either the clavicle or scapula, depending on the reconstruction. The muscle is tensioned to resting tension with the arm in adduction for deltoid reconstructions, flexion for triceps reconstructions, and extension for biceps reconstructions.

Postoperatively, patients were kept in an abduction sling or functional brace, depending on tumor location, for 10–12 weeks. Patients then began neuromuscular retraining of the latissimus.

## 3. Results

### 3.1. Functional Outcomes

At the most recent follow-up, the mean elbow and shoulder ranges of motion were 105° (range 0–130°) and 150° (range 0–180°, Table 2), respectively. The mean Musculoskeletal Tumor Society score was 88% (range, 37–100%). The mean muscle strength score was 4 (range 0–5).

The functional outcomes of patient nine are outliers and worthy of note. This patient sustained a radiation-associated humerus fracture 5 years postoperatively, which resulted in poor functional outcomes. However, prior to this injury, their elbow and shoulder ranges of motion were 100° and 175°, respectively, and their strength was 5/5.

### 3.2. Oncologic Outcomes

**Figure 3:**
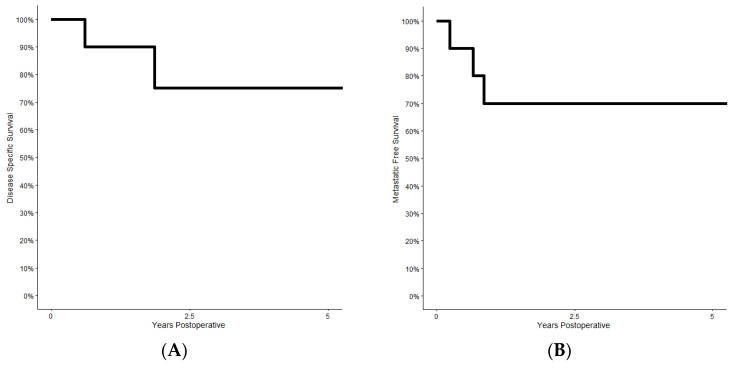
Following resection and reconstruction of the upper extremity with a pedicled latissimus flap, the 5 year disease-specific (**A**) and metastatic-free survival (**B**) were 75% and 70%, respectively.

Following resection, the 5 year disease-specific survival and metastatic free survival were 75% and 70%, respectively (Figure 3). No patients experienced local recurrence. Four patients developed metastatic disease, and three patients died of the disease.

### 3.3. Complications

Six patients experienced complications, five of whom were in the immediate post-operative period. Four patients had a recipient site complication; all were secondary to delayed wound healing. Two of these patients (patients three and four) required negative pressure wound vacuum therapy and staged split-thickness skin grafting. One additional patient (patient eight) had a donor site seroma which required irrigation and debridement of the wound. Complications resulted in three additional surgeries, including the split-thickness skin grafting procedures, and an irrigation, and a debridement procedure. There were no total flap losses. As previously mentioned, patient nine sustained a radiation-associated humerus fracture 5 years postoperatively. This was treated non-operatively due to progressive metastatic disease.

## 4. Discussion

Limb salvage is the preferred method of operative management of STS in the upper extremity, but the extent of resection required to achieve negative margins can commonly impart a substantial functional limitation. The utilization of free and pedicled flaps for limb salvage of upper extremity STS is well documented (Table 3), whereas functional latissimus flaps following STS resection have not been well described or analyzed. The results of the current study emphasize the utility of these flaps to provide both soft tissue coverage and to restore function in patients after sarcoma resection.

The multidisciplinary oncologic team is critical to the successful management of patients with soft tissue sarcoma [15,22,23]. At our institution, the orthopedic oncology team works in conjunction with plastic surgery, radiology, pathology, radiation oncology, medical oncology, and physical therapy to ensure patients are given the best chance at disease-free survival and to optimize functional outcomes. For operative management, the orthopedic oncology and plastic surgery teams evaluate the patient preoperatively in the clinic setting to determine the combined surgical plans. This allows the reconstructive team to plan for the extent of resection and appropriately counsel the patient on the expected outcome, and has been shown to improve the care of patients undergoing resection of a soft-tissue sarcoma in the setting of prior radiotherapy [18,19].

Reconstruction of the upper extremity utilizing a pedicled or a free flap following sarcoma resection has been shown to reduce disability and restore function [19]. In a large series of patients undergoing upper extremity reconstruction, Payne et al. [19] determined there was no difference in the complication profile between the use of a free or pedicled flap and recommended the use of either flap based on an individualized approach to the patient, as both forms of reconstruction were found to restore form and function to the upper extremity. Although there are free muscle flap options to restore elbow function [6,24], we feel the ease of flap elevation and the location of the latissimus make it an ideal option to restore function to the upper extremity.

The latissimus, works synergistically with other muscles of the shoulder and arm to execute shoulder adduction, extension, and internal rotation [15,25,26,27]. The extent of functional impairment following harvesting the flap is debated, and patients should be cautioned on the potential for a clinically important decrease in shoulder strength and discomfort, which could limit activities of daily living. That being said, there is currently a lack of other options to provide a functional reconstruction following these resections. Following resection of the deltoid, triceps, or biceps, patients will be limited in shoulder and/or elbow range of motion, which leads to functional impairment and impacts activities of daily living [20,21,28,29]. As such, the utilization of a functional latissimus flap allows for restoration of function of the arm; as such, the benefits of the use of these flaps likely outweigh the potential complications, as shown in the outcomes of the current study (MSTS score of 88%, strength of 4/5, and elbow and shoulder range of motion of 105° and 150°).

Triceps reconstruction has been debated, as previously it was thought elbow flexion was more important than active extension; however, Ozaniak et al. [21] found that active elbow extension is essential for many activities of daily living. If the latissimus cannot be used to reconstruct the triceps, synthetic options are available; however, these are expensive and can be difficult to acquire [30]. An additional option is the use of a fascia lata autograft, which has been shown to restore elbow extension following sarcoma resection [31]. In the case report by Clancy et al. [31], the authors utilized a strip of fascia lata to reconstruct the triceps insertion in a patient with a soft-tissue sarcoma of the distal arm. This restored function, however, required an additional flap for coverage of the wound [31]. One of the benefits of the pedicled latissimus flap is its ability to not only restore function but also provide wound coverage; as such, this is our preferred reconstructive technique for these patients.

There were multiple early complications secondary to wound healing complications. While radiation therapy improves rates of local control, it is not a benign intervention and is associated with the complications observed in our study [4,32,33,34,35]. Although consideration for postoperative radiotherapy can be given, the permanent functional complications of stiffness and fibrosis, especially around joints, occur secondary to the higher treatment doses and volumes [36,37]. In addition, preoperative radiotherapy allows for a planned close margin of resection around critical structures [38,39,40,41,42,43], preserving the neurovasculature of the upper extremity and ultimate hand function. With this, our preferred treatment algorithm is preoperative radiotherapy followed by a planned close margin of resection with immediate reconstruction. Patients should be cautioned about the risk of wound healing complications; however, the results of the study show that these can be salvaged without having a substantial impact on upper extremity function or the overall patient outcome.

This study is not without limitations. This is a retrospective review of a relatively small group of patients who underwent soft tissue sarcoma resection and reconstruction with a functional pedicled latissimus at a single institution. This limits the analysis we are able to perform and precludes statistical analyses. Each oncologic resection was different based on the anatomic factors of the tumor, and as such, each reconstruction was different based on the individualized needs of the patient. However, the consistency of the single multidisciplinary orthopedic oncologic team allows for the surgical plans and executions to be somewhat uniform, the nature of single institution studies diminishes generalizability.

## 5. Conclusions

Although early complications were common in patients undergoing functional pedicled latissimus reconstruction of the biceps, triceps, and/or deltoid, this reconstructive technique allowed for wound coverage, potential space obliteration, and restoration of function in the upper extremity following resection of large soft tissue sarcomas. This series demonstrates the functional outcomes for this technique, and a multidisciplinary team approach between the orthopedic oncology and plastic surgery teams to taking care of these patients affords them optimal outcomes.

## Figures and Tables

**Table 1:** Patients undergoing functional pedicled latissimus flap reconstruction.

Patient	Age	Gender	Location	Neurovascular Resection	Tumor Size	Tumor Depth	Histology	Additional Treatments
1	61	Female	Long Head Triceps Lateral Triceps Medial Triceps	None	5.6 cm	Deep	UPS	Preop RT (50 Gy)
2	76	Female	Middle Deltoid Posterior Deltoid	Middle and Anterior Axillary Nerve	5.1 cm	Deep	UPS	Preop RT (50 Gy)
3	56	Male	Long Head Biceps Short Head Biceps Brachialis	MSC Nerve	Re-Excision	Superficial	Myxofibro	Preop RT (50 Gy)
4	60	Female	Long Head Biceps Short Head Biceps Brachialis	MSC Nerve	Re-Excision	Deep	Extraskeletal Osteosarcoma	Preop RT (50 Gy)
5	26	Male	Long Head Triceps Lateral Triceps Medial Triceps	None	11.8 cm	Deep	NTRK Sarcoma	Preop RT (50 Gy)
6	77	Male	Long Head Triceps Lateral Triceps Medial Triceps	None	10.8 cm	Deep	Pleomorphic Liposarcoma	Preop RT (50 Gy)
7	66	Male	Long Head Biceps Short Head Biceps Brachialis Anterior Deltoid Coracobrachialis Pectoralis Major	MSC Nerve	4 nodules 1.5–7.5 cm	Deep	Pleomorphic Spindle Cell Sarcoma	Preop RT (50 Gy)
8	68	Male	Anterior Deltoid Middle Deltoid Posterior Deltoid	Middle and Anterior Axillary Nerve	10.3 cm	Deep	Myxofibro	Preop RT (50 Gy)
9	63	Male	Long Head Biceps Short Head Biceps Brachialis Anterior Deltoid Coracobrachialis Brachioradialis	MSC Nerve	24 cm	Deep	UPS	Preop RT (50 Gy)
10	77	Male	Long Head Triceps Lateral Triceps Medial Triceps	None	12.6 cm	Deep	UPS	Preop RT (50 Gy) Intraop RT (11 Gy)

UPS: Undifferentiated Pleomorphic Sarcoma; Preop: preoperative; RT: Radiotherapy; MSC: Musculocutaneous; Myxofibro: Myxofibrosarcoma; NTRK: Neurotrophic receptor tyrosine kinase; Intraop: intraoperative.

**Table 2:** Outcomes Following Reconstruction.

Patient	Elbow ROM	Shoulder ROM	Strength	MSTS93	Complication	Tumor Recurrence	Clinical Status
1	10–120°	0–170°	4/5	93%	-	-	ANED 4 Years
2	0–130°	0–180°	5/5	97%	Delayed Healing	-	ANED 4 Years
3	10–120°	0–160°	5/5	100%	Delayed Healing Needed STSG	-	ANED 1 Year
4	10–110°	0–170°	4/5	93%	Delayed Healing Needed STSG	-	ANED 2.5 Years
5	0–130°	0–180°	4/5	90%	-	-	ANED 2 Years
6	0–120°	0–170°	4/5	97%	-	-	ANED 11 years
7	10–115°	0–170°	5/5	97%	Delayed Healing	Metastatic Disease Lymph nodes, Lungs	AWED 1 year
8	0–130°	0–150°	4/5	93%	Donor Site Seroma Needed I and D	Metastatic Disease Lungs	DOD 2 years
9	0°	0°	0/5	37%	Radiation Associated Humerus Fracture	Metastatic Disease Lungs	DOD 8 Years
10	25–120°	0–160°	3/5	80%	-	Metastatic Disease Soft Tissue, Lungs	DOD 7 Months

MSTS: Musculoskeletal Tumor Society score; ROM: Range of motion.

**Table 3:** Previous series examining the use of latissimus flaps for sarcoma reconstruction.

Citation	Patients	Tumor Location	Surgical Technique Data	Outcomes
Grinsell et al. [6]	Twenty-two patients with neoadjuvant radiotherapy for soft-tissue sarcoma. Free flap reconstruction.	Seventeen, lower extremity. Three, upper extremity. Two, abdominal wall.	Innervated muscle flap reconstructions. Latissimus dorsi was utilized in seven patients. Two upper extremity latissimus cases.	Six postoperative complications related to the reconstruction. Upper extremity had QuickDASH scores of 0 and 31.
Lucattelli et al. [7]	Systematic review of 17 studies including 132 patients with an average age of 49.25 years and 53% male.	-	Functional reconstruction of defects of the shoulder, arm, forearm, and hand. Multiple flap types used.	Flap success rate of nearly 100%.
Kapoor et al. [9]	One hundred and twenty-seven patients. Mean age of 58 in the pedicle flap group and 51 in the free flap group. One hundred and thirty-four men and forty-four women are included.	Thirty-nine, upper extremity. One hundred and thirty-nine, lower extremity.	Thirty-nine patients with upper extremity sarcomas, twenty-seven were reconstructed with a pedicle flap, and twelve were reconstructed with a free flap.	Pedicle flap patients had higher rates of donor site complications. Free flaps had higher recipient site complications. Mean QuickDASH score for upper extremity reconstruction was 5.98.
Rednam et al. [12]	Case report of an 8-year-old patient requiring a functional latissimus flap.	-	Posterior compartment of the leg reconstructed with a myocutaneous latissimus dorsi free flap.	Ability to bear full weight. Four/five plantarflexion strength after the innervated latissimus flap reconstruction.
Houdek et al. [15]	Twelve patients with soft tissue sarcoma of the thigh. Nine males and three females. Mean age of 56 ± 12 years.	Soft tissue sarcomas of the quadriceps, hamstrings, or both.	Reconstruction with a free functional latissimus flap for the quadriceps or hamstrings.	The mean knee range of motion (ROM) was 89 ± 24°. MSTS93 score was 90 ± 15%. Muscle strength was 4 ± 1. Nine of the twelve patients ambulated without gait aids. Seven (58%) patients sustained a complication.
Chan et al. [18]	One hundred and seventeen patients underwent one hundred and twenty-two treatments in total. Preoperative radiotherapy, tumor resection, and flap reconstruction.	Any soft tissue sarcomas and other locally aggressive soft tissue tumors.	Variety of free and pedicled flaps utilized. Ten pedicled latissimus dorsi flaps utilized in this cohort.	Twenty-five percent of patients experienced a major wound complication. Seventeen patients required further surgery. No difference in complications between pedicled and free flaps.
Payne et al. [19]	One hundred and thirteen patients underwent limb-preserving STS resection of the upper extremity, requiring a free or pedicled-flap.	Soft tissue sarcomas of the upper extremity.	Seventy-seven cases that utilized a pedicled flap. Thirty-six cases which utilized a free flap. Most common pedicled flap was the latissimus dorsi flap. Most common free flap was the anterolateral thigh flap.	Twenty-six complications among the cohort. Wound infection (twelve) and delayed healing (seven). No differences in complication rates between pedicled vs. free flaps.
Muramatus et al. [20]	Four patients with sarcoma of the deltoid. All were male. Mean age 68 years old.	Wide surgical margin involving the entire deltoid muscle with the axillary nerve.	Reconstructed with ipsilateral pedicled latissimus flaps.	No flap losses or other wound complications. Active abduction was >160° in all four patients. Average MSTS score was 92%.
Ozaniak et al. [21]	Case report of a 47-year-old male.	Recurrence of the malignant peripheral nerve sheath tumor in the posterior compartment of the arm.	Resection of the triceps brachii followed by a pedicled functional latissimus flap.	The patient did not experience any complications. His elbow range of motion was 15–130 degrees six months postoperatively.

## Data Availability

The data presented in this study are available on request from the corresponding author. The data are not publicly available due to ethical restrictions.
